# Predicting Prostate Biopsy Outcomes: A Preliminary Investigation on Screening with Ultrahigh B-Value Diffusion-Weighted Imaging as an Innovative Diagnostic Biomarker

**DOI:** 10.1371/journal.pone.0151176

**Published:** 2016-03-10

**Authors:** Kun Zhang, Yanguang Shen, Xu Zhang, Lu Ma, Haiyi Wang, Ningyu An, Aitao Guo, Huiyi Ye

**Affiliations:** 1 Department of Radiology, PLA General Hospital, Beijing, China; 2 Department of Radiology, Navy General Hospital, Beijing, China; 3 Department of Urology, PLA General Hospital, Beijing, China; 4 Department of Pathology, PLA General Hospital, Beijing, China; Shenzhen institutes of advanced technology, CHINA

## Abstract

**Background:**

Routine screening of prostate specific antigen (PSA) is no longer recommended because of a high rate of over-diagnosis of prostate cancer (PCa).

**Objective:**

To evaluate the efficacy of diffusion-weighted magnetic resonance imaging (DW-MRI) for PCa detection, and to explore the clinical utility of ultrahigh b-value DW-MRI in predicting prostate biopsy outcomes.

**Methodology:**

73 male patients were selected for the study. They underwent 3T MRI using T2WI conventional DW-MRI with b-value 1000 s/mm^2^, and ultrahigh b-value DW-MRI with b-values of 2000 s/mm^2^ and 3000 s/mm^2^. Two radiologists evaluated individual prostate gland images on a 5-point rating scale using PI-RADS, for the purpose of region-specific comparisons among modalities. Sensitivity, specificity, accuracy, positive predictive value (PPV), negative predictive value (NPV) and likelihood ratios (LR) were investigated for each MRI modality. The area under the receiver operating characteristic (ROC) curve (AUC) was also calculated.

**Results:**

Results showed the improved diagnostic value of ultrahigh b-value DWI-MRI for detection of PCa when compared to other b values and conventional MRI protocols. Sensitivity values for 3000 s/mm^2^ in both peripheral zone (PZ) and transition zone (TZ) were significantly higher than those observed with conventional DW-MRI—Specificity values for 3000 s/mm^2^ in the TZ were significantly higher than other b-value images, whereas specificity values using 3000 s/mm^2^ in the PZ were not significantly higher than 2000 s/mm^2^ images. PPV and NPV between 3000 s/mm^2^ and the other three modalities were significantly higher for both PZ and TZ images. The PLRs and NLRs of b-value 3000 s/mm^2^ DW-MRI in the PZ and TZ were also recorded. ROC analysis showed greater AUCs for the b value 3000 s/mm^2^ DWI than for the other three modalities.

**Conclusions:**

DW-MRI with a b-value of 3000 s/mm^2^ was found to be the most accurate and reliable MRI modality for PCa tumor detection and localization, particularly for TZ lesion discrimination. It may be stated that the b-value of 3000 s/mm^2^ is a novel, improved diagnostic biomarker with greater predictive accuracy for PCa prior to biopsy.

## Introduction

Prostate cancer (PCa) is the second most commonly diagnosed cancer worldwide, accounting for about one-quarter of newly diagnosed cases in males [1,2]. The steady increase of incidence rates in PCa is largely due to widespread prostate-specific antigen (PSA) testing. However, routine screening for PSA is no longer recommended, because it is associated with a high rate of overdiagnosis [3–5]. Despite its low specificity for diagnosing PCa, PSA screening still remains the most frequently used tool for this purpose [6].The PSA test yields a positive predictive value of 25.1%, with a range of 17.0% to 57.0% [7]. Ultrasound (US)-guided systematic transrectal biopsy is the current reference standard for diagnosing prostate lesions; this invasive procedure however, requires specific expertise [8].

Diffusion-weighted magnetic resonance imaging (DW-MRI) is increasingly being used to study the abdomen and pelvis, and more specifically, the prostate [9,10]. DW-MRI is performed without administering contrast medium, and requires less time than other functional MRI techniques [11]. In clinical diagnosis, the b-value is the essential parameter that affects PCa detection capability, but the normal prostate signal intensity is often not suppressed in DWI, despite using b-values of approximately 1000 s/mm^2^ [12]. A higher b-value DWI would be more advantageous for highlighting the contrast between cancer signal intensity and normal tissue, because of greater diffusivity and less T2 shine-through effect. Earlier studies [13–16] indicate that ultrahigh b-value DWI improved the diagnostic accuracy for PCa detection, when compared to a standard b-value of 600–1000 s/mm^2^. At present, there is no consensus on the optimal b value for PCa diagnosis using 3T MRI, and the effect of an ultrahigh b-value of 3000 s/mm^2^ remains unclear.

The present study investigates the diagnostic accuracy of ultrahigh b-value DW-MRI at 3 T, with the aid of the Prostate Imaging Reporting and Data System: version 1(PI-RADSv1) scoring system, and US-guided systematic transrectal biopsies. The study’s objective was to explore whether ultrahigh b-value imaging could be the diagnostic biomarker in predicting prostate biopsy outcomes.

## Materials and Methods

### Patients

All procedures were performed in accordance with the ethical standards of the Declaration of Helsinki. The study was approved by the PLA General Hospital review board. Written informed consent was obtained from each patient prior to MRI examination. Between February 2014 and January 2015, 103 male patients who were consecutively subjected to routine screening for PCa were enrolled from a single institution. The patients’ medical history was reviewed, and they underwent a physical examination (including digital rectal examination (DRE)), and a prostate specific antigen (PSA) test. The inclusion criteria were:

- Age: ≥55 years
- Absence of lower urinary tract symptoms: this was indicated by the International Prostate Symptom Score (IPSS) being equal to zero
- Ability and willingness to sign the form for informed consent;
- Absence of contraindications to MRI examination
- A physical condition that would tolerate MRI and biopsy
- No evidence of PCa or any other malignancy
- No history of urinary tract surgery (e.g., laser, microwave, radiofrequency ablation, stents)
- No congenital malformations
- An interval of three weeks between the MRI examination and biopsy

Of the 103 patients originally listed, thirty were ineligible for the investigation because of the following reasons (*n* indicates number of patients):

- Incidence of lower urinary tract symptoms and IPSS score > 0 (*n* = 17)
- intolerance to MRI examination (*n* = 1)
- Refusal to undergo biopsy (*n* = 2)
- History of previous biopsy within three months prior to MRI examination (*n* = 3)
- Prevalence of radiotherapy, chemotherapy or other treatment of prostate disease (*n* = 7)

Finally, 73 patients were found to be eligible for inclusion in the present investigation. After the MRI examination, all patients underwent routine US-guided transrectal.prostate biopsies involving 12 cores. No MRI-targeted biopsies were performed.

### Magnetic Resonance Imaging Protocol

MRI examinations were conducted using a 3-T MRI system (Discovery 750, GE Healthcare, Milwaukee, WI, USA). Patients were imaged in the supine position using a surface phased-array coil. Endorectal coil was not employed. Conventional MRI scan sequences included both axial fast spin-echo T1-weighted images (T1WI), and T2-weighted images (T2WI). T2WI of the entire prostate and seminal vesicles was performed in the axial, coronal, and sagittal planes by means of a fat-suppressed fast spin-echo sequence. Conventional DW-MRI with b-values of 0 and 1000 s/mm^2^ was also performed in the axial plane. The ultrahigh b-value DW-MRI was performed with b-values of 2000 s/mm^2^ and 3000 s/mm^2^ in the axial plane. Images were obtained by using a single-shot spin-echo planar sequence. Both axial T2WI and DW-MRI images were obtained at the same slice location (Table 1). Dynamic contrast enhanced (DCE) was excluded from the MRI protocols as DCE is not part of scanning protocols for prostate imaging in this study.

**Table 1:** Magnetic resonance imaging acquisition parameters.

Protocol	Sequence	TR	TE	FA	S/I	FOV	Matrix
T2WI	FR FSE	4079	96	90	4.0/0.5	16 ×16	288 × 256
DW-MRI							
b = 1000 s/mm^2^	SE EPI	5000	73	90	4.0/0.5	32 ×32	160 × 160
b = 2000 s/mm^2^	SE EPI	5000	73	90	4.0/0.5	32 ×32	160 × 160
b = 3000 s/mm^2^	SE EPI	5000	73	90	4.0/0.5	32 ×32	160 × 160

Key: DW-MRI—diffusion-weighted magnetic resonance imaging; FA—flip angle; FOV—field of view; FR FSE—fast recovery fast spin echo; SE EPI—spin-echo echo-planar imaging; S/I—section thickness/intersection gap; TE = echo time; TR = repetition time

### Measurement of Prostate Lesions

#### By imaging

Interpretation of images obtained from matched T2WI, conventional DW-MRI, and ultrahigh b-value DW-MRI of the prostate, was done by two radiologists with 3 years (L.M.) and 10 years (K.Z.) experience respectively. They were blinded to clinical data. All lesions were rated on a scale from 1 to 5, based on PI-RADS, in accordance with the European Society of Urogenital Radiology guidelines for prostate MRI (Table 2) [17].

**Table 2:** Evaluation of each magnetic resonance imaging sequence according to the Prostate Imaging Reporting and Data System score.

Score	T2WI for the PZ	T2WI for the TZ	DWI
1	Uniform high signal intensity	Heterogeneous TZ adenoma with well-defined margins	No reduction in ADC. No increase in SI on any high b-value image (≥800)
2	Linear, wedge-shaped, or geographic areas of lower signal intensity, usually not well demarcated	Areas of more homogeneous low signal intensity; however well marginated, originating from the TZ/BPH	Diffuse, hypersignal intensity on ≥800 image with low ADC.No focal features (linear, triangular or geographic features are allowed)
3	Intermediate appearances	Intermediate appearances	Intermediate appearances
4	Discrete, homogeneous low signal focus/mass confined to the prostate	Ill-defined areas of more homogeneous low signal intensity	Focal areas of reduced ADC. Isointense SI on high b-value images (≥800)
5	Same as 4 plus extra-capsular extension/invasive behavior or mass effect on the capsule (bulging),or broad (>1.5 cm) contact with the surface	Same as 4 plus involving the anterior fibromuscular stroma or the anterior horn of the PZ, usually lenticular or water-drop shaped	Focal area/mass of reduced ADC. Hypersignal intensity on the high b-value images (≥800)

Key: ADC—apparent diffusion coefficient; BPH—benign prostatic hyperplasia; DWI—diffusion-weighted imaging; PZ—peripheral zone; SI—signal intensity; T2WI–T2-weighted imaging; TZ—transition zone.

#### By biopsy

**Fig 1:**
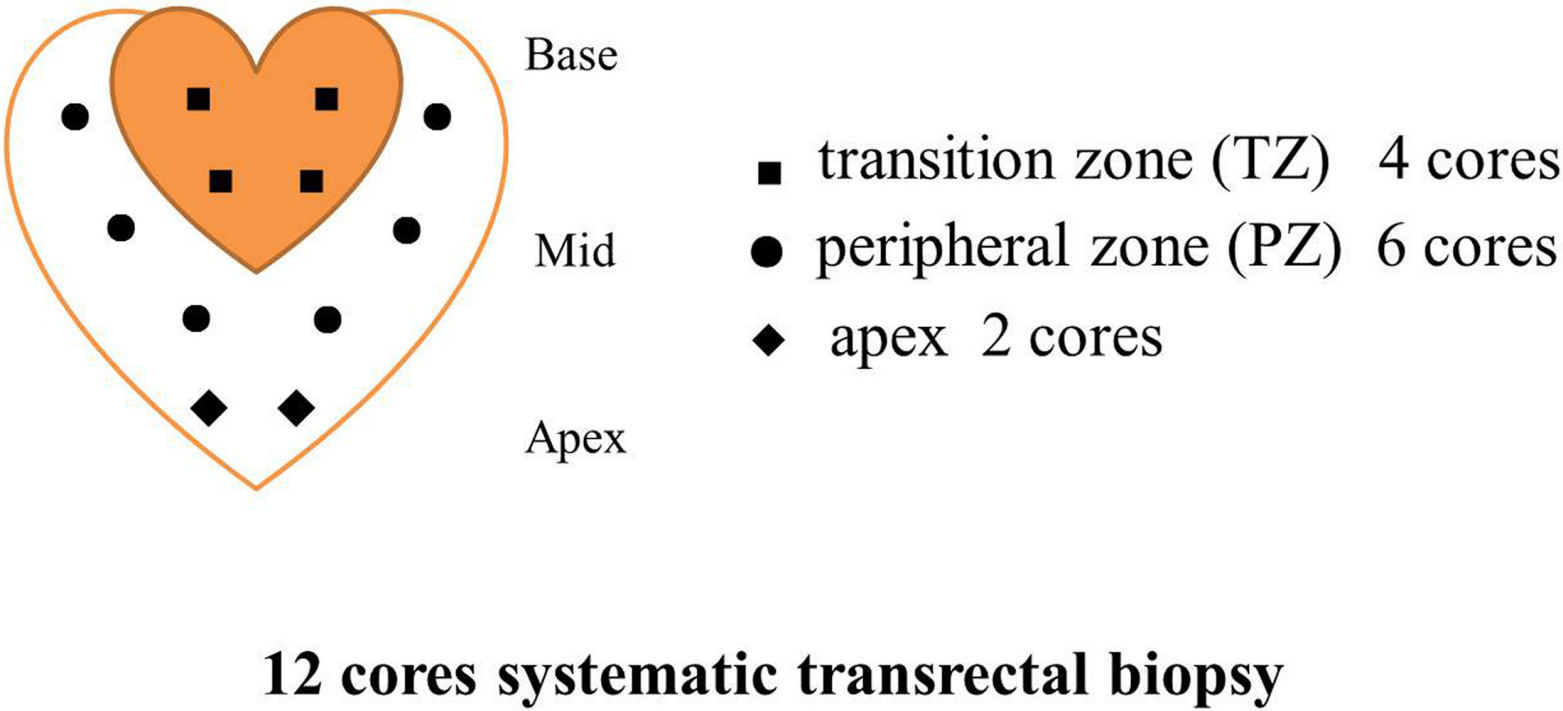
Diagram showing 12 cores systematic transrectal biopsy locations.

Ultrasound-guided systematic transrectal biopsies were performed by two urologists with 8 and 10 years experience, on all the sample patients in one single institution, using the same instruments and methodology. The urologists were aware of the MRI results at the time of biopsy. 12 prostate gland specimens were obtained from each patient undergoing biopsy: 6 from the peripheral zone [PZ], 4 from the transition zone [TZ], and 2 from the apex (Fig 1). Region specific comparisons among the modalities were made by dividing the prostate gland into 12 regions: bilateral PZs (including the upper, middle and lower regions), bilateral TZs (including the upper and lower regions), and bilateral apex.

Evaluation of results was carried out separately on a 5-point PI-RADS rating scale for the PZs and the TZs. The obtained results were dichotomized into numerical scores. Scores of 1 and 2 were regarded as indicating absence of cancer (normal or benign lesions), while scores of 3–5 pointed to the presence of cancer (abnormal indicative of malignant lesions) [18,19].

### Statistical Analysis

Continuous variables were presented as median and range, while categorical variables were presented as percentages. Descriptive statistics for the patient, and parameters such as PSA levels, MRI, and histopathologic characteristics, were calculated for the study sample of 73 males. The 141 unique lesions identified through MRIs (99 in PZ and 42 in TZ) were compared with the help of localization and Gleason scores. The reference standard was judged by systematic evaluation of biopsy results: regions in biopsy specimens showing pathological evidence of cancer were considered as positive, whereas regions showing non-presence of cancer were considered as negative. This pattern of analysis was repeated for each prostate region including PZ and TZ. Sensitivity, specificity, positive predictive value (PPV), negative predictive value (NPV), negative likelihood ratios (NLRs), and positive likelihood ratios (PLRs) were calculated for each image modality. Differences between T2WI and three DW-MRI b-values (0 and 1000 s/mm^2^, 0 and 2000 s/mm^2^, and 0 and 3000 s/mm^2^), were analyzed using the McNemar test. Corroboration of observations made by the two readers, was achieved with the *kappa* coefficient. Values falling between 0.41–0.60, 0.61–0.80, and above 0.81, were respectively regarded as being in moderate, substantial or perfect agreement. Receiver operating characteristic (ROC) analysis was applied to each MRI modality. The areas under the curves (AUCs) were compared by using the *Z* test. Statistical analysis was performed using IBM SPSS Statistics v.19 for Windows (IBM Corp, Armonk, NY, USA). Statistical significance was defined at a *p* value<0.05.

## Results

### Patient Characteristics

Table 3 summarizes patient characteristics. PSA levels and pathological findings were calculated for the 73 sample patients. 141 unique lesions (PZ: 99; TZ: 42) were identified by performing biopsies.

**Table 3:** Patient characteristics.

Characteristics	Patients with cancer	Patients without cancer
Number	54	19
Age (yrs)	67 (55–81)	70 (60–84)
PSA (ng/ml)		
<4	1 (2)	1 (5)
4–10	25 (46)	12 (63)
10–20	15 (28)	6 (32)
>20	13 (24)	0 (0)
Gleason score		
3 + 3 = 6	16 (30)	NA
3 + 4 = 7	9 (16)	NA
4 + 3 = 7	13 (24)	NA
≥8	16 (30)	NA

Key: NA—not applicable; PSA—prostate specific antigen

Data is presented as median (interquartile range) for continuous variables, and numerical value (percentage) for categorical variables

### Inter-Reader Agreement

Based on the PI-RADS score between the two radiologists (readers), the inter-reader agreement was found to be almost perfect for the ultrahigh b-value DW-MRI of 3000 s/mm^2^ (*kappa* values 0.81 in PZ, 0.855 in TZ). Agreement was substantial for the ultrahigh b-value DW-MRI of 2000 s/mm^2^ (*kappa* values 0.731 in PZ, 0.754 in TZ), and moderate for the conventional DW-MRI of 1000 s/mm^2^ (*kappa* values 0.554 in PZ, 0.568 in TZ) as well as T2WI (*kappa* values 0.523 in PZ, 0.612 in TZ).

### Diagnostic Value of Magnetic Resonance Imaging

#### Peripheral zone lesions

**Fig 2:**
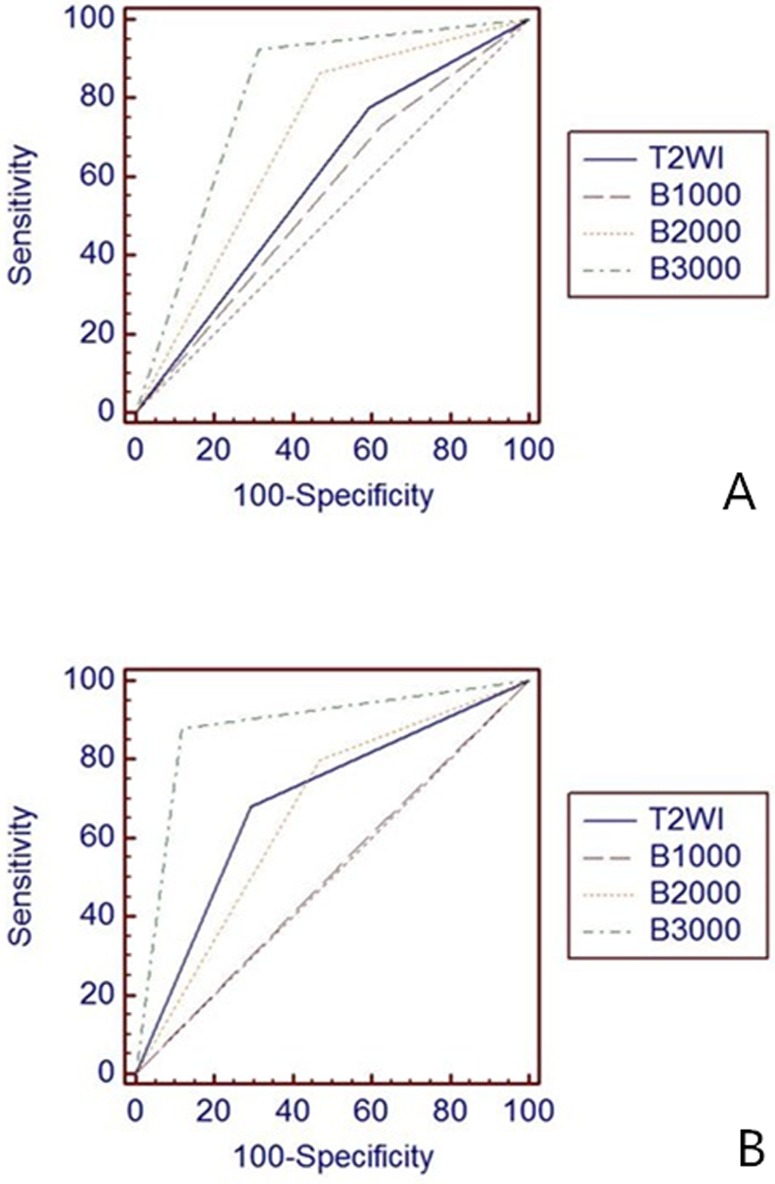
ROC curves for diagnosis in peripheral zone (A) and transition zone (B).

141 lesions were identified in the prostate glands of the 73 sample patients. With respect to MRI analysis of 99 lesions in the peripheral zone (PZ), 65 (65.7%), 61 (61.6%), 76 (76.8%), and 84 (84.8%) were diagnosed accurately with the T2WI, conventional DW-MRI and ultrahigh b-value DW-MRI respectively. Of the 42 lesions in the transition zone (TZ), 29 (69%), 22 (52.4%), 29 (69%), and 37 (88.1%) were correctly diagnosed. Table 4 summarizes the sensitivity, specificity, PPV, NPV, PLR, and NLR for each MRI modality. The ROC curves and AUCs are displayed in Fig 2 and Table 5.

**Table 4:** Estimates of sensitivity, specificity, accuracy, positive predictive value and negative predictive value per modality for both peripheral zone and transition zone.

	**Sensitivity**			**Specificity**			**PPV**		**NPV**		
	**PZ**	**TZ**		**PZ**		**TZ**	**PZ**	**TZ**	**PZ**		**TZ**
T2WI	0.776 (0.655–0.865)	0.680 (0.464–0.842)		0.406 (0.242–0.592)		0.706 (0.440–0.886)	0.732 (0.612–0.827)	0.772 (0.542–0.913)	0.464 (0.280–0.658)		0.600 (0.364–0.800)
b1000	0.731 (0.607–0.829)	0.600 (0.389–0.782)		0.375 (0.217–0.563)		0.412 (0.194–0.665)	0.710 (0.587–0.810)	0.600 (0.389–0.782)	0.400 (0.232–0.592)		0.412 (0.194–0.665)
b2000	0.881 (0.773–0.943)	0.800 (0.587–0.924)		0.531 (0.350–0.705)		0.529 (0.285–0.761)	0.797 (0.685–0.878)	0.714 (0.511–0.860)	0.680 (0.464–0.843)		0.643 (0.356–0.860)
b3000	0.925 (0.827–0.972)	0.880 (0.677–0.968)		0.688 (0.499–0.833)		0.882 (0.623–0.980)	0.861 (0.755–0.928)	0.917 (0.715–0.985)	0.815 (0.613–0.930)		0.833 (0.577–0.956)
	**PLR (C)**			**NLR (C)**			**PLR (W)**		**NLR (W)**		
	**PZ**		**TZ**	**PZ**	**TZ**		**PZ**	**TZ**	**PZ**	**TZ**	
T2WI	1.307 (0.955–1.790)		2.31 (1.056–5.064)	0.551 (0.323–0.941)	0.453 (0.244–0.842)		2.737 (1.817–4.123)	3.400 (1.523–7.591)	1.154 (0.754–1.766)	0.667 (0.365–1.217)	
b1000	1.170 (0.862–1.588)		1.020 (0.612–1.699)	0.717 (0.436–1.778)	0.971 (0.510–1.851)		2.450 (1.644–3.651)	1.500 (0.842–2.671)	1.500 (1.015–2.216)	1.429 (0.814–2.507)	
b2000	1.879 (1.286–2.745)		1.700 (0.990–2.920)	0.225 (0.111–0.455)	0.378 (0.156–0.917)		3.933 (2.468–6.270)	2.500 (1.330–4.698)	0.471 (0.256–0.865)	0.556 (0.255–1.213)	
b3000	2.961 (1.763–4.973)		7.480 (2.019–27.718)	0.109 (0.045–0.259)	0.136 (0.046–0.399)		6.200 (3.462–11.102)	11.000 (2.902–41.690)	0.227 (0.101–0.510)	0.200 (0.070–0.574))	

Key: (C)- conventional; NLR—negative likelihood ratios; NPV—negative predictive value; PLR—positive likelihood ratio;.PPV—positive predictive value; PZ—peripheral zone; TZ—transition zone; (W)—weighted by prevalence.

Numbers are expressed as percentages. Numbers in parentheses are 95% confidence intervals.

**Table 5:** Modality wise areas under the curves (AUCs) for both peripheral zone and transition zone.

	T2WI		b1000		b2000		b3000	
	PZ	TZ	PZ	TZ	PZ	TZ	PZ	TZ
AUCs	0.591	0.693	0.553	0.506	0.698	0.665	0.806	0.881
95%CI	0.488–0.689	0.532–0.826	0.450–0.653	0.347–0.663	0.598–0.787	0.503–0.803	0.715–0.879	0.744–0.960

In the peripheral zone, the sensitivity for ultrahigh b-value DW-MRI was 92.5% (95% confidence interval [CI]), 82.7–97.2% with a b-value of 3000 s/mm^2^, 88.1% (95% CI), and 77.3–94.3% for 2000 s/mm^2^. These values were significantly higher than those observed for T2WI (77.6% [95% CI, 65.5–86.5]; *p*<0.05), and conventional DW-MRI with a b-value of 1000 s/mm^2^ (73.1% [95% CI, 60.7–83.9]; *p*<0.05). The detection of lesions in the PZ was comparable with ultrahigh b-value DW-MRI at 3000 s/mm^2^ and 2000 s/mm^2^ (*p* = 0.249). Specificity was found to be 68.8% (95% CI, 49.9–83.3) with b-value 3000 s/mm^2^ images; 40.6% (95% CI, 24.2–59.2; *p*< 0.05) for T2WI, and 37.5% (95% CI, 21.7–56.3; *p*<0.05) for conventional DW-MRI at 1000 s/mm^2^. With respect to the PZ, these values were not significantly higher than b-value 2000 s/mm^2^ images (53.1% [95% CI, 35.0–70.5]; *p* = 0.074).

#### Transition zone lesions

In the TZ, sensitivity values at 3000 s/mm^2^ were significantly higher than those observed with conventional DW-MRI (88.0% [95% CI, 67.7–96.8] *vs* 60.0% [95% CI, 38.9–78.2]; *p*<0.05). Comparatively, the values did not reach statistical significance with images produced with b-value of 2000 s/mm^2^ (*p* = 0.480). However, the specificity value of 88.2% (95% CI, 62.3–98.0) was significantly higher statistically at 3000 s/mm^2^, when compared to other b-value images (*p*<0.05) in the TZ.

#### Reduction in false-positive rate

The false-positive rate of ultra-high-b-value DWI was reduced by 15.7%-31.3% in PZ, and 17.6%-47.0% in TZ. In other words, 15%–47% patients could have safely avoided biopsy by examination with b value of 3000 s/mm^2^ ultra-high-b-value DWI.

#### Other parameters

Figures for PPV obtained with b-value of 3000 s/mm^2^ as well as conventional DW-MRI were significantly higher, with respect to both PZ (0.861) and TZ (0.917); the same was true for NPV (PZ: 0.815; TZ: 0.833). The PLRs and NLRs of b-value 3000 s/mm^2^ DW-MRI were 6.2 and 0.227 respectively in the PZ, and 11 and 0.2 respectively in the TZ. ROC analysis showed greater AUCs for the b value of 3000 s/mm^2^ DWI, than for the other three modalities.

**Fig 3:**
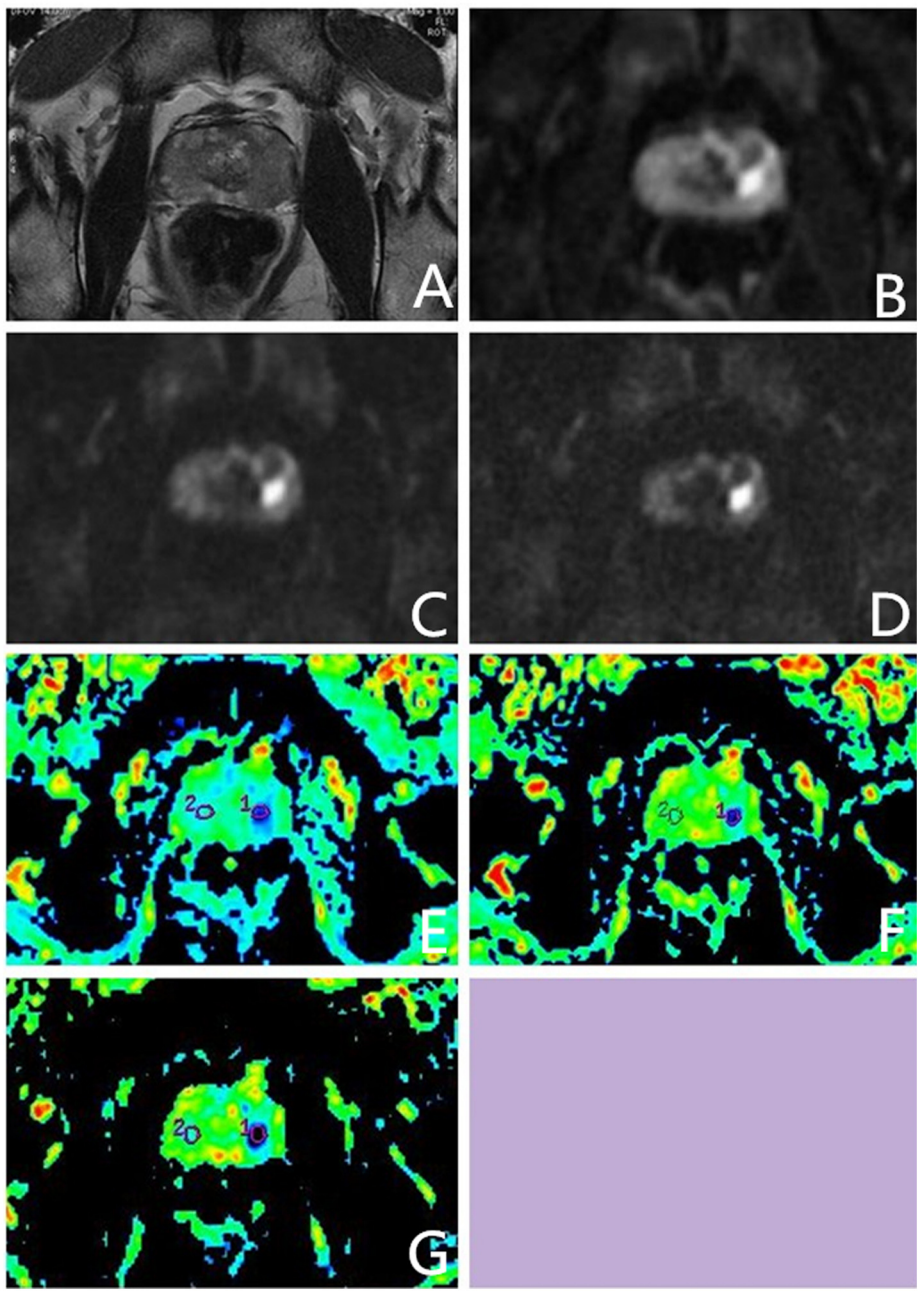
Images from a 55 yr old male patient with total prostate-specific antigen (PSA) 3.41 ng/ml and free PSA 0.247ng/ml. Systematic transrectal biopsy confirmed. prostate cancer (Gleason score 4 + 3 = 7). (A) High-solution T2-weighted imaging shows low signal intensity area in the left boundary region of the transition zone (TZ) and peripheral zone (PZ); (B–D) On ultrahigh b-value diffusion-weighted imaging (DWI) (b = 1000, 2000, 3000 s/mm^2^), lesions on the left showed significantly higher signal nodules. Increase in b-values reduced normal tissue signal intensity, but not signals from prostate cancer tissue; (E–G) With conventional and ultrahigh b-value DWI (b = 1000, 2000, 3000 s/mm^2^), apparent diffusion coefficient map (ADC), and organization of lesions on the left and normal ADC values were significantly different; lesion ADC values decreased with increase in b-value. Key: ADC—apparent diffusion coefficient; DWI—diffusion-weighted imaging; PSA—prostate-specific antigen; PZ—peripheral zone; TZ—transition zone.

**Fig 4:**
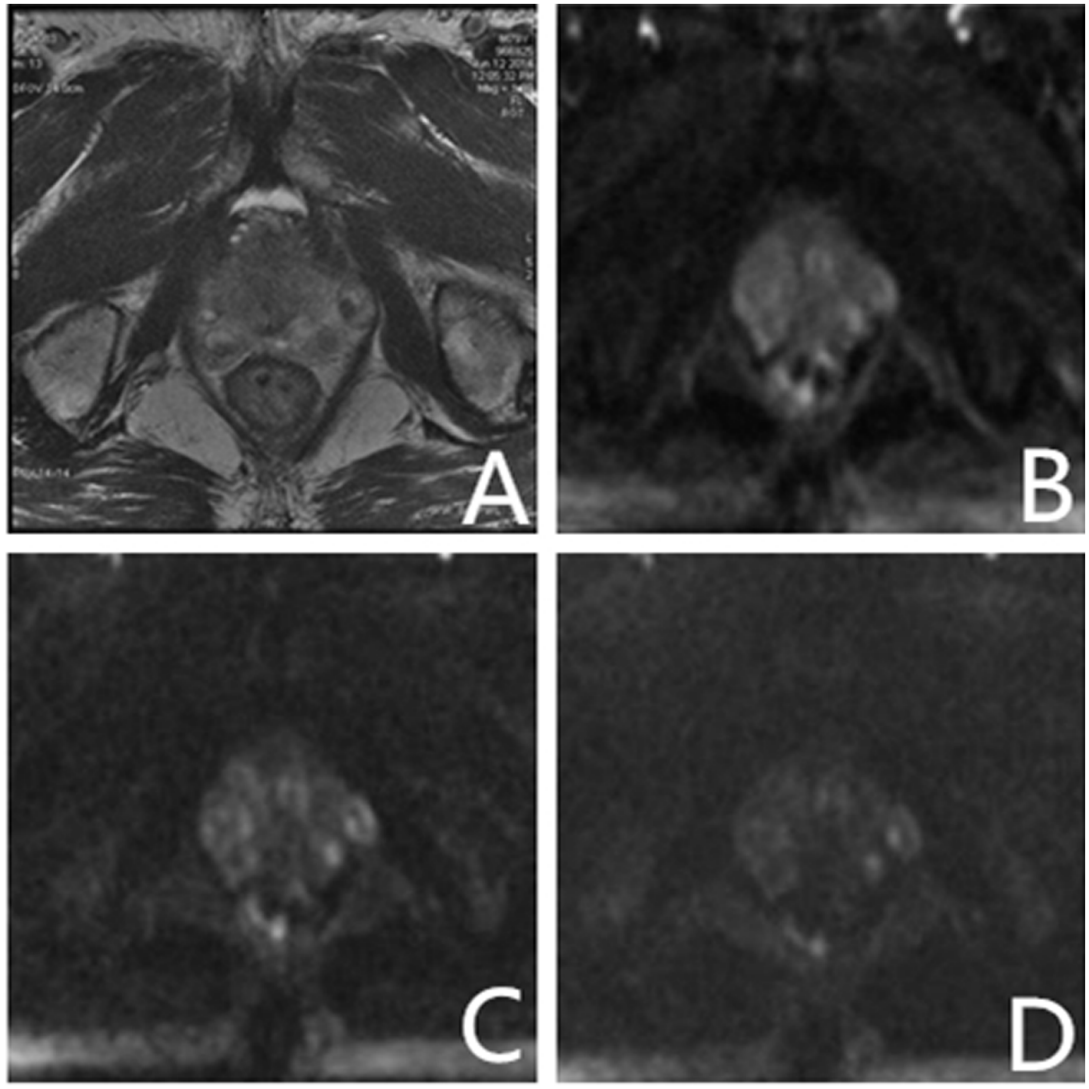
Images from a 79 yr old male patient with total PSA 6.1 ng/ml. Systematic transrectal biopsy confirmed prostate hyperplasia and prostatitis. (A) High-solution T2-weighted imaging shows low signal lesions on bilateral peripheral zone; (B–D) On conventional and ultrahigh b-value diffusion-weighted imaging (b = 1000, 2000, 3000 s/mm^2^), bilateral PZ lesions showed slightly higher signal nodules. Intensity of lesion signals decreased with increase in b-value, whereas the intensity of normal tissue signals reduced significantly. Key: PSA—Prostate specific antigen; PZ—Peripheral zone.

**Fig 5:**
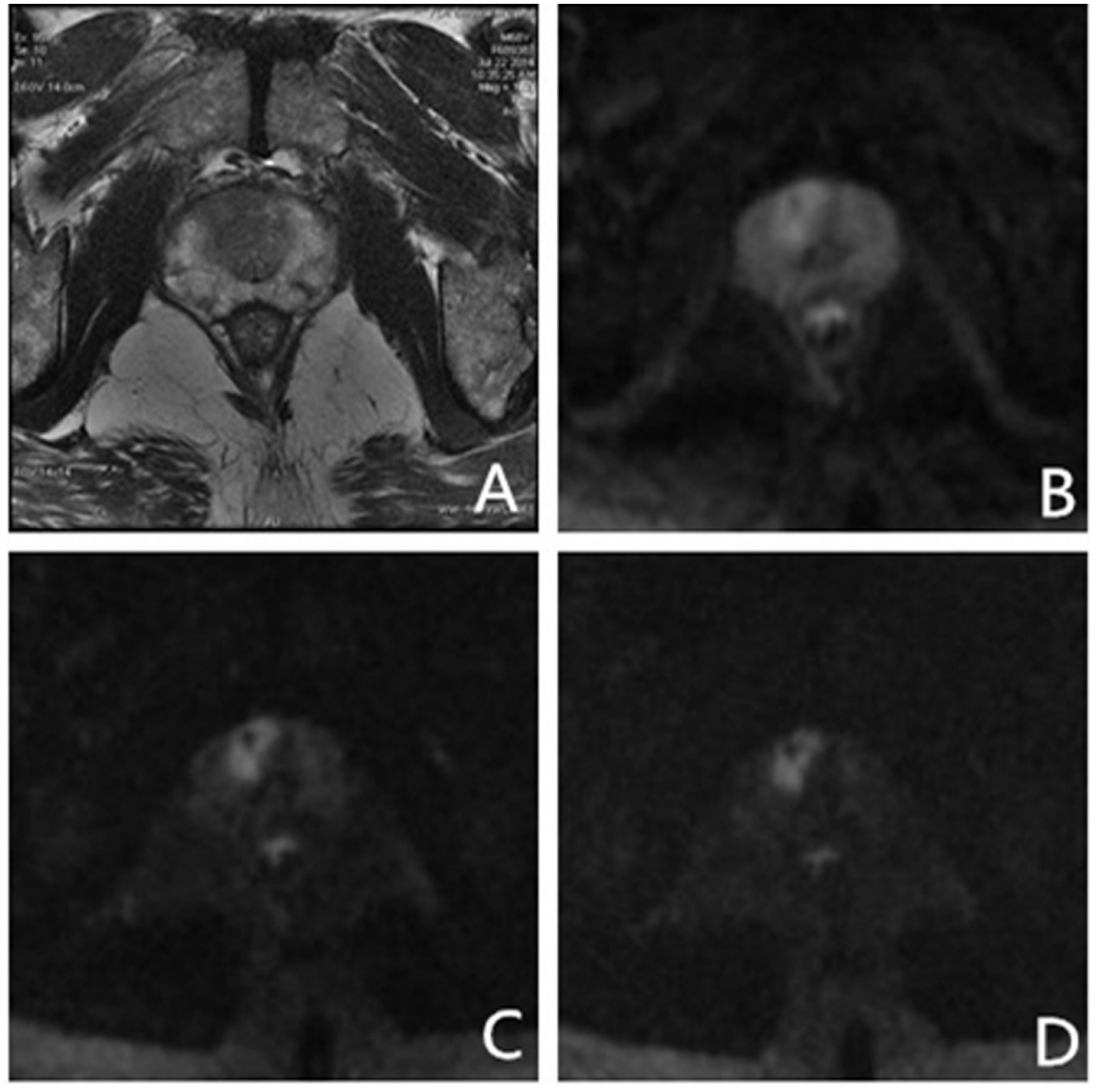
Images of a 68 yr old male patient with total PSA 10.5 ng/ml and free PSA 1.66 ng/ml. Systematic transrectal biopsy confirmed PCa (Gleason score 4 + 3 = 7). (A) High-solution T2-weighted imaging shows low signal intensity area in the right TZ, and PI-RADS score of 2 due to subtle mass effect; (B) PI-RADS score of 3 on DWI with b-value 1000 s/mm^2^. The cancer is ambiguous because of high signal intensity from surrounding parenchyma; (C, D) PI-RADS score of 5 on ultrahigh DWI with b-value 2000, 3000 s/mm^2^. High signal intensity areas in the right portion of the TZ are clearly visible. Key: PSA—Prostate specific antigen; PCa—prostate cancer; TZ—transition zone; DWI—diffusion-weighted imaging; PI-RADS—Prostate Imaging Reporting and Data System.

In an exceptional case, a male patient aged 55 years, with normal serum PSA levels, exhibited no prostate-related clinical symptoms. The ultrahigh b-value DW-MRI however, showed a suspicious lesion in the left middle region of the PZ. US-guided systematic transrectal biopsies revealed PCa with Gleason score 4 + 3 = 7 (Fig 3). Two more cases were presented as follows (Figs 4 and 5).

## Discussion

Multiparametric MRI is a tool vital to PCa diagnosis, and DW-MRI has the advantages of being both free from contrast medium, and requiring less time than other MRI techniques [11, 20–22]. At present, the added value of dynamic contrast-enhanced MR imaging, which is acquired with intravenous administration of contrast agent, is not well established. Enhancement alone is not definitive for diagnosing prostate cancer, and absence of early enhancement does not exclude the possibility of being a cancerous lesion [23]. Furthermore, most published data show that the diagnostic value of DCE is modest when compared to combination of T2W and DWI [23,24].The present study explored the efficacy of ultrahigh b-value DW-MRI in predicting the outcome of prostate biopsy. The results demonstrated that for detecting prostate lesions, ultrahigh b-value DW-MRI at b-values of 2000 s/mm^2^ and 3000 s/mm^2^, were superior to conventional DW-MRI of 1000 s/mm^2^ imaging at 3 T. Similar studies have showed that b-value of 2000 s/mm^2^ DW-MRI outperformed 800 s/mm^2^ or 1000 s/mm^2^ DW-MRI at 3 T [13,14]. To the best of our knowledge, the present study is the first of its kind to show the advantage of using ultrahigh b-value (3000 s/mm^2^) DW-MRI for detecting PCa at 3 T.

Advances in technology have promoted the rapid growth of non-invasive methods to detect PCa. A higher b-value DW-MRI essentially provides a greater contrast between cancer and non-cancer tissue. Some earlier studies have tried to evaluate the PCa diagnostic capability of DW-MRI with an ultrahigh b-value >1000 s/mm^2^ [15,16]. The present study differs from that of Kim *et al* [15] in that the latter reported greater accuracy with 1000 s/mm^2^ DW-MRI in PCa localization, compared to 2000 s/mm^2^ at 3 T. Kim *et al* applied a relatively long TE (83–85 ms for 1000 s/mm^2^, 93–95 ms for 2000 s/mm^2^), whereas the present study used a shorter TE of DWI (73 ms for 1000, 2000 and 3000 s/mm^2^) [15]. The limitation of the Kim *et al* study [15] is that a relatively longer TE is indicative of more time for T2 dephasing, thereby causing a reduction of the signal-to-noise ratio (SNR). Furthermore, a study of DW-MRI, acquired with the same shorter TE for different b-values, supports the advantage of ultrahigh b-value DW-MRI [13].

Results of the present study were elicited from each prostate region. Detection of cancerous and non-cancerous lesions in the PZ, was achieved with the help of 2000 s/mm^2^ultrahigh b-value DW-MRI, resulting in significant improvement in specificity, PPV, and NPV. These results agree with those of Ueno *et al* [13]. However, these researchers [13] could not identify any significant improvement in the TZ. Cancers as pinpointing the location are difficult in this region because of the high signal intensity of normal parenchyma or benign prostate hyperplasia [16,25]. This is different from the features exhibited by cancers located in the PZ. It is important to distinguish TZ cancers accurately with imaging, in order to facilitate biopsy. Some research investigations have reported poorer diagnostic performance in the TZ for T2WI plus conventional DWI, and T2WI alone [13,14,16]. To overcome the limitations of high signal emitted by the prostate itself on DWI with b-value 1000 s/mm^2^, Katahira *et al* [16] used 2000 s/mm^2^ DWI that showed significantly better sensitivity, specificity, accuracy, PPV, and NPV at 1.5 T. Results of the Katahira *et al* study are in agreement with outcomes of the present study. However, 3 T was applied because of increased SNR using ultrahigh b-value DW-MRI at 2000 and 3000 s/mm^2^. The present investigation revealed that the b-value of 3000 s/mm^2^ markedly improved sensitivity, specificity, PPV, and NPV; moreover, there was substantial enhancement of PLR and NLR with a b-value of 2000 s/mm^2^. The ultrahigh b-value DW-MRI at 3000 s/mm^2^ had the additional advantage of detection in both the TZ and PZ, particularly in the former.

In the present study, the ultrahigh b-value DW-MRI showed outstanding diagnostic performance in the sub-group, with the 3000 s/mm^2^ DW-MRI alone outdoing both T2WI and conventional DW-MRI, thus implying that the ultrahigh b-value DW-MRI alone could serve as a promising biomarker for guiding the prostate biopsy decision. In the present study, a man with normal serum PSA was found to have PCa when subjected to examination with the ultrahigh b-value DW-MRI. This finding demonstrates that, especially at 3000 s/mm^2^, ultrahigh b-value DW-MRI seems to be the most sensitive and specific MRI modality for tumor detection and localization. Predictive values were significantly higher for both the PZ and TZ. Ultrahigh b-value DW-MRI was identified as a vital and independent predictor of PCa at the stage of prostate biopsy.

The present investigation has both advantages and limitations. Firstly, imaging was performed at 3 T with the advantage of a higher SNR, namely, improved spatial resolution and different imaging sequences [26]. Secondly, for region-specific comparisons corresponding to the results, the PPV and NPV of ultrahigh b-value DW-MRI increased respectively by 8.7–15.1% in 2000 s/mm^2^ and 28–41.5% in 3000 s/mm^2^, when compared to conventional DW-MRI in the PZ, and 9.9–30.2% and 20.5–39.5%, respectively, in the TZ.

A potential limitation of the present research effort is that all the patients were from a single center, therefore, external validation is needed through further studies. Another limitation is that lesions were determined by systematic biopsy. MRI findings do not correspond one by one to the biopsy results, because lesion-guided biopsies were not conducted. Nevertheless, attempts were made to minimize errors in the study through careful region-specific comparisons. Thirdly, we did not compare the multiparametric MRI in the diagnosis of prostate cancer. However, that was not the purpose of this study. Whether the effect of ultrahigh b-value DW-MRI could improve the grading and staging of PCa, still remains to be established through rigorous scientific evaluation.

## Conclusion

It may be concluded that DW-MRI with a b-value of 3000 s/mm^2^is the most accurate and reliable MRI modality for PCa tumor detection and localization, particularly in the case of TZ lesion discrimination. It is superior to T2WI, and conventional DW-MRI in distinguishing patients with a high probability of PCa, when undergoing prostate biopsy. These results suggest that ultrahigh b-value DW-MRI, based on PI-RADS, helps radiologists and urologists to reach consensus on clinical decision making for prostate biopsy. It is expected to be a novel diagnostic biomarker that improves predictive accuracy for PCa. Further exploration of the value of ultrahigh b-value DW-MRI is warranted at a multicenter level.

## Supporting Information

S1 Table Patient characteristics and PI-RADS scores. (XLSX)

S2 Table Diagnostic test (2x2 table) of T2WI and DWI with b-value 1000 s/mm^2^ in PZ. (DOCX)

S3 Table Diagnostic test (2x2 table) of DWI with b-value 1000 s/mm^2^ and ultrahigh DWI with b-value 2000 s/mm^2^ in TZ. (DOCX)

S4 Table Comparison of ROC curves in PZ. (DOCX)

S5 Table Comparison of ROC curves in TZ. (DOCX)
