# Supplementary material for: Predicting Prostate Biopsy Outcomes: A Preliminary Investigation on Screening with Ultrahigh B-Value Diffusion-Weighted Imaging as an Innovative Diagnostic Biomarker
Source: PLoS One. 2016 Mar 10;11(3):e0151176. doi: 10.1371/journal.pone.0151176 (PMC4786278; doi:10.1371/journal.pone.0151176)
Supplement: S2 Table — (DOCX) [file pone.0151176.s002.docx]

**Table S2.Diagnostic test (2x2 table) of T2WI and DWI with b-value 1000 s/mm^2^ in PZ.**

1. **PZ-T2WI**

|  | Condition | | Totals |
| --- | --- | --- | --- |
|  | Absent | Present |  |
| Test Positive | 19 | 52 | 71 |
| Test Negative | 13 | 15 | 28 |
| Totals | 32 | 67 | 99 |

1. **PZ-b-value 1000 s/mm^2^**

|  | Condition | | Totals |
| --- | --- | --- | --- |
|  | Absent | Present |  |
| Test Positive | 20 | 49 | 69 |
| Test Negative | 12 | 18 | 30 |
| Totals | 32 | 67 | 99 |
