# Supplementary material for: Predicting Prostate Biopsy Outcomes: A Preliminary Investigation on Screening with Ultrahigh B-Value Diffusion-Weighted Imaging as an Innovative Diagnostic Biomarker
Source: PLoS One. 2016 Mar 10;11(3):e0151176. doi: 10.1371/journal.pone.0151176 (PMC4786278; doi:10.1371/journal.pone.0151176)
Supplement: S3 Table — (DOCX) [file pone.0151176.s003.docx]

**Table S3. Diagnostic test (2x2 table) of DWI with b-value 1000 s/mm^2^ and ultrahigh DWI with b-value 2000 s/mm^2^ in TZ.**

1. **TZ- b-value 1000 s/mm2**

|  | Condition | | Totals |
| --- | --- | --- | --- |
|  | Absent | Present |  |
| Test Positive | 10 | 15 | 25 |
| Test Negative | 7 | 10 | 17 |
| Totals | 17 | 25 | 42 |

1. **TZ- b-value 2000 s/mm2**

|  | Condition | | Totals |
| --- | --- | --- | --- |
|  | Absent | Present |  |
| Test Positive | 8 | 20 | 28 |
| Test Negative | 9 | 5 | 14 |
| Totals | 17 | 25 | 42 |
