# Supplementary material for: Predicting Prostate Biopsy Outcomes: A Preliminary Investigation on Screening with Ultrahigh B-Value Diffusion-Weighted Imaging as an Innovative Diagnostic Biomarker
Source: PLoS One. 2016 Mar 10;11(3):e0151176. doi: 10.1371/journal.pone.0151176 (PMC4786278; doi:10.1371/journal.pone.0151176)
Supplement: S4 Table — (DOCX) [file pone.0151176.s004.docx]

**Table S4. Comparison of ROC curves in PZ**

| Variable 1 | T2WI |
| --- | --- |
| Variable 2 | B1000 |
| Variable 3 | B2000 |
| Variable 4 | B3000 |
| Classification variable | P |

| Sample size |  | 99 |
| --- | --- | --- |
| Positive group : | P = 1 | 67 |
| Negative group : | P = 0 | 32 |

|  | AUC | SE ^a^ | 95% CI ^b^ |
| --- | --- | --- | --- |
| T2WI | 0.591 | 0.0510 | 0.488 to 0.689 |
| B1000 | 0.553 | 0.0513 | 0.450 to 0.653 |
| B2000 | 0.698 | 0.0495 | 0.598 to 0.787 |
| B3000 | 0.806 | 0.0447 | 0.715 to 0.879 |

^a^ DeLong et al., 1988

^b^ Binomial exact

**Pairwise comparison of ROC curves**

| T2WI ~ B1000 | |
| --- | --- |
| Difference between areas | 0.0380 |
| Standard Error ^c^ | 0.0202 |
| 95% Confidence Interval | -0.00149 to 0.0775 |
| z statistic | 1.886 |
| Significance level | P = 0.0593 |
| T2WI ~ B2000 | |
| Difference between areas | 0.107 |
| Standard Error ^c^ | 0.0345 |
| 95% Confidence Interval | 0.0396 to 0.175 |
| z statistic | 3.109 |
| Significance level | P = 0.0019 |
| T2WI ~ B3000 | |
| Difference between areas | 0.215 |
| Standard Error ^c^ | 0.0459 |
| 95% Confidence Interval | 0.125 to 0.305 |
| z statistic | 4.685 |
| Significance level | P < 0.0001 |
| B1000 ~ B2000 | |
| Difference between areas | 0.145 |
| Standard Error ^c^ | 0.0388 |
| 95% Confidence Interval | 0.0693 to 0.221 |
| z statistic | 3.747 |
| Significance level | P = 0.0002 |
| B1000 ~ B3000 | |
| Difference between areas | 0.253 |
| Standard Error ^c^ | 0.0482 |
| 95% Confidence Interval | 0.159 to 0.348 |
| z statistic | 5.253 |
| Significance level | P < 0.0001 |
| B2000 ~ B3000 | |
| Difference between areas | 0.108 |
| Standard Error ^c^ | 0.0357 |
| 95% Confidence Interval | 0.0380 to 0.178 |
| z statistic | 3.023 |
| Significance level | P = 0.0025 |

^c^ DeLong et al., 1988
