# Supplementary material for: Predicting Prostate Biopsy Outcomes: A Preliminary Investigation on Screening with Ultrahigh B-Value Diffusion-Weighted Imaging as an Innovative Diagnostic Biomarker
Source: PLoS One. 2016 Mar 10;11(3):e0151176. doi: 10.1371/journal.pone.0151176 (PMC4786278; doi:10.1371/journal.pone.0151176)
Supplement: S5 Table — (DOCX) [file pone.0151176.s005.docx]

**Table S5. Comparison of ROC curves in TZ.**

| Variable 1 | T2WI |
| --- | --- |
| Variable 2 | B1000 |
| Variable 3 | B2000 |
| Variable 4 | B3000 |
| Classification variable | P |

| Sample size |  | 42 |
| --- | --- | --- |
| Positive group : | P = 1 | 25 |
| Negative group : | P = 0 | 17 |

|  | AUC | SE ^a^ | 95% CI ^b^ |
| --- | --- | --- | --- |
| T2WI | 0.693 | 0.0742 | 0.532 to 0.826 |
| B1000 | 0.506 | 0.0793 | 0.347 to 0.663 |
| B2000 | 0.665 | 0.0746 | 0.503 to 0.803 |
| B3000 | 0.881 | 0.0522 | 0.744 to 0.960 |

^a^ DeLong et al., 1988

^b^ Binomial exact

**Pairwise comparison of ROC curves**

| T2WI ~ B1000 | |
| --- | --- |
| Difference between areas | 0.187 |
| Standard Error ^c^ | 0.0633 |
| 95% Confidence Interval | 0.0629 to 0.311 |
| z statistic | 2.954 |
| Significance level | P = 0.0031 |
| T2WI ~ B2000 | |
| Difference between areas | 0.0282 |
| Standard Error ^c^ | 0.0581 |
| 95% Confidence Interval | -0.0856 to 0.142 |
| z statistic | 0.486 |
| Significance level | P = 0.6267 |
| T2WI ~ B3000 | |
| Difference between areas | 0.188 |
| Standard Error ^c^ | 0.0627 |
| 95% Confidence Interval | 0.0652 to 0.311 |
| z statistic | 3.000 |
| Significance level | P = 0.0027 |
| B1000 ~ B2000 | |
| Difference between areas | 0.159 |
| Standard Error ^c^ | 0.0573 |
| 95% Confidence Interval | 0.0464 to 0.271 |
| z statistic | 2.770 |
| Significance level | P = 0.0056 |
| B1000 ~ B3000 | |
| Difference between areas | 0.375 |
| Standard Error ^c^ | 0.0774 |
| 95% Confidence Interval | 0.224 to 0.527 |
| z statistic | 4.848 |
| Significance level | P < 0.0001 |
| B2000 ~ B3000 | |
| Difference between areas | 0.216 |
| Standard Error ^c^ | 0.0658 |
| 95% Confidence Interval | 0.0874 to 0.346 |
| z statistic | 3.288 |
| Significance level | P = 0.0010 |

^c^ DeLong et al., 1988
